# Riboflavin: The Health Benefits of a Forgotten Natural Vitamin

**DOI:** 10.3390/ijms21030950

**Published:** 2020-01-31

**Authors:** Nittiya Suwannasom, Ijad Kao, Axel Pruß, Radostina Georgieva, Hans Bäumler

**Affiliations:** 1 Institute of Transfusion Medicine, Center of Tumor Medicine, Charité—Universitätsmedizin Berlin, Charitéplatz 1, 10117 Berlin, Germany; nittiya.suwannasom@charite.de (N.S.); ijad.kao@charite.de (I.K.); axel.pruss@charite.de (A.P.); radostina.georgieva@charite.de (R.G.); 2 School of Medical Sciences, University of Phayao, Phayao 56000, Thailand; 3 Biophysics and Radiology, Department of Medical Physics, Faculty of Medicine, Trakia University, 6000 Stara Zagora, Bulgaria

**Keywords:** riboflavin, vitamin B2, complementary medicine, functional food, oxidative stress, cancer, neurodegeneration, muscular degeneration

## Abstract

Riboflavin (RF) is a water-soluble member of the B-vitamin family. Sufficient dietary and supplemental RF intake appears to have a protective effect on various medical conditions such as sepsis, ischemia etc., while it also contributes to the reduction in the risk of some forms of cancer in humans. These biological effects of RF have been widely studied for their anti-oxidant, anti-aging, anti-inflammatory, anti-nociceptive and anti-cancer properties. Moreover, the combination of RF and other compounds or drugs can have a wide variety of effects and protective properties, and diminish the toxic effect of drugs in several treatments. Research has been done in order to review the latest findings about the link between RF and different clinical aberrations. Since further studies have been published in this field, it is appropriate to consider a re-evaluation of the importance of RF in terms of its beneficial properties.

## 1. Introduction

Riboflavin (RF) was first documented in 1879 by Blythto as a yellow pigment found in milk [1]. RF, chemically, is 7, 8-dimethyl-10-ribityl-isoalloxazine which consists of a flavin isoalloxazine ring bound to a sugar side chain, Ribitol [2]. RF is also known as an essential vitamin B_2_, a water-soluble vitamin, is heat stable. Cooking does not lower levels of RF, however exposure to light could destroy it. RF can be found in a wide variety of foods and natural sources, especially milk, organ meats—mostly in calf liver, egg, fish, nuts, certain fruits and legumes, wild rice, mushrooms, dark green leafy vegetables, yeast, beer, cheese and dietary products [1,3]. RF is poorly stored by vertebrates because of its limited absorption in humans. Therefore, orally supplied RF by a healthy diet is required to avoid ariboflavinosis which causes cheilitis, sore tongue, and a scaly rash on the scrotum or vulva. RF causes no known toxicity, since at higher intakes it is excreted in the urine and not stored [3]. RF is found in different concentrations in various human body fluids and organs (Table 1). Recent analytical procedures developed for detection of RF in biological samples were described in several reviews [4,5,6].

**Figure 1:**
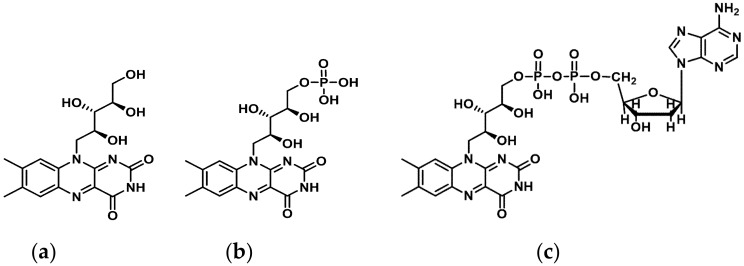
Structure of (**a**) Riboflavin (RF), (**b**) Flavin monophosphate (FMN), and (**c**) Flavin adenine dinucleotide (FAD). ChemDraw (PerkinElmer Informatics, Inc. MA, USA).

RF is phosphorylated intracellular to flavin mononucleotide (FMN) and further metabolized to flavin adenine dinucleotide (FAD) (Figure 1).

**Figure 2:**
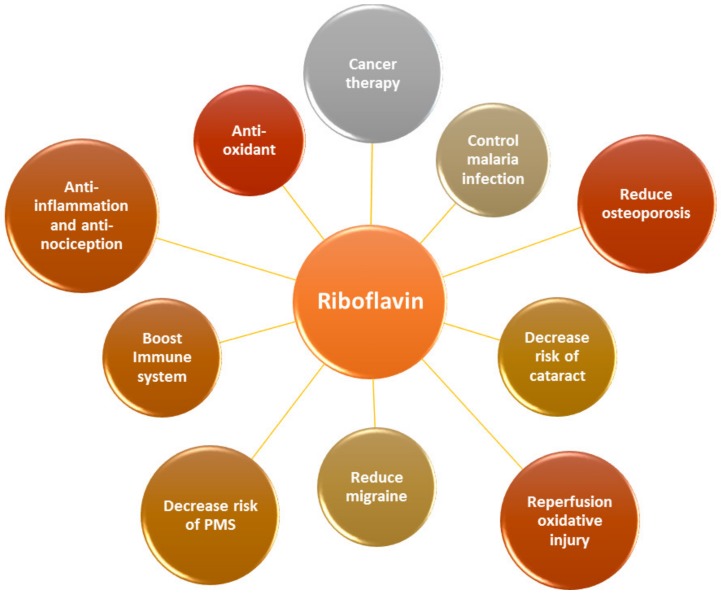
RF is an essential vitamin for multiple physiological aspects in the body.

Both FMN and FAD play a key role as cofactors in energy metabolism and are required for co-enzyme function in numerous oxidation and reduction reactions in all aerobic forms of life. RF has been used in dietary supplements and for inflammatory disease treatments such as angulus infectious, cheilitis, glossitis [7], sepsis [8,9], cataracts and migraine headaches [3] (Figure 2).

## 2. Beneficial Health Effects of RF

### 2.1. Antioxidant Properties

Oxidative stress plays a crucial functional role in the pathogenesis of various human disease states including ischemia-reperfusion injury, diabetes and angina pectoris [10]. Oxidative stress is a key effect of aging and degenerative diseases along with aging. The authors have demonstrated that supplementation of RF significantly extended the lifetime and strengthened the reproduction of fruit flies via enhancing the activity of antioxidant enzymes [11]. RF also activates the synthesis of a normal extracellular matrix and reduces reactive oxygen species (ROS) levels in keratoconus [12]. The evidence of the effect of RF and its roles for reactive oxygen species in various symptoms and diseases is summarized in Table 2.

RF was used for its potent antioxidant and anti-inflammatory effects in the ischaemic liver, protecting hepatic parenchymal cells against I/R injury [13].

**Figure 3:**
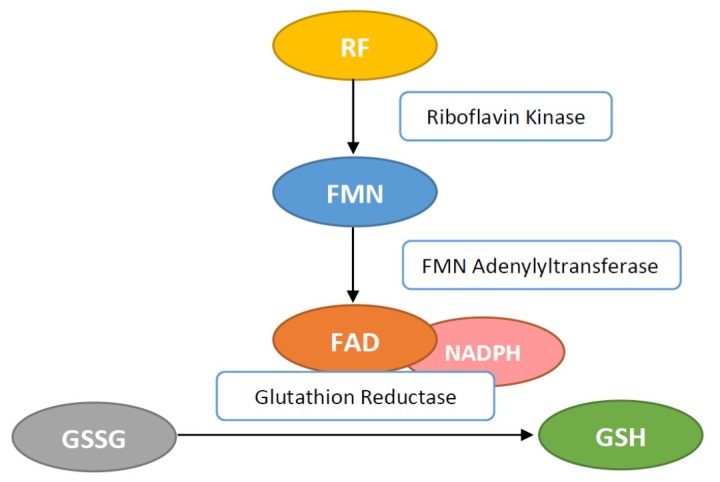
Flavin adenine dinucleotide (FAD) activates the glutathione reductase (GR) by transferring hydrogen for conversion of glutathione disulfide (GSSG) to glutathione (GSH).

Other antioxidant enzymes concentrations—like superoxide dismutase (SOD), catalase and glutathione peroxidase—are also influenced by RF concentration. RF plays an important role for the antioxidant status inside cell systems as well as being part of the glutathione reductase (GR) and xanthine oxidase system. RF in the form of FAD is necessary for GR enzyme to convert oxidized glutathione (GSSG) to the reduced glutathione (GSH) (Figure 3). It then functions as an endogenous antioxidant in different cells [14].

### 2.2. Reperfusion Oxidative Injury

Reperfusion oxidative injuries refer to the tissue damages which occur after ischemia when free radicals and inflammatory cytokines are increased (Table 3). RF can attenuate oxidative injuries through its ability to scavenge free radicals and therefore decrease re-oxygenation injuries. New evidence suggests that ROS have a crucial role in the pathogenesis of ischemia/reperfusion injury. Studies in various cell cultures and animal species have shown a variety of protective actions of RF in many organs, e.g., the neuroprotective effect of cerebral ischemia [15]. The authors showed that pre-treatment of the SH-SY5Y cells with RF before oxygen glucose deprivation (OGD) experiments significantly reduced the OGD-induced lactate dehydrogenase (LDH) release and significantly increased cell viability. The cell viability and LDH secretion were quantified in the SH-SY5Y cell line and cortical neuron cultures using the cell counting Kit-8 and the LDH quantification kit compared to that treated-OGD group. In vivo study showed a significant neuroprotective activity via the modulation of the miR-203/c-Jun signalling pathway [15].

**Table 2:** Evidence on effect of RF and its roles for reactive oxygen species in various symptoms and diseases. Upregulation ↑, downregulation ↓.

Model	Dose	Antioxidant Enzymes	Key Findings	References
Anti-aging in Drosophila melanogaster (fruit fly)	RF at 120 µg/mL	SOD1 ↑; CAT ↑; lipofuscin (LF) ↓	RF prolonged the life span and increased reproductive capacity through anti-oxidative stress pathway involving enhancing the activity of SOD1 and CAT and inhibiting lipofuscin accumulation	[11]
Keratoconus corneal stroma cells	Keratoconus cells were treated with low dose of RF at 0.167 µg/mL	Increasing gene expression of antioxidant enzymes: aldehyde dehydrogenase 3A1, CAT, enolase 1, GPx 1, haem oxygenase 1, SOD 1 and transketolase	RF improved the synthesis of a normal extracellular matrix and downregulated ROS level in keratoconus. It was quatified by the total collagen protein in the keratoconic stroma.	[12]
Diabetes-induced cardiac dysfunction	RF at 20 mg/kg was treated after streptozotocin-induced diabetes type I.	SOD↑, MDA↓, HO-1 protein level↑	RFK can reduced the risk of cardiac dysfunction by increasing antioxidant, HO-1 and decreasing CTGF levels as well as improving lipid profile	[16]
Diabetes mellitus type-2	RF at 10 and 20 mg/kg was treated after alloxan-induced DM	SOD↑, catalase↑, GSH↑, MDA↓	Decreased pancreatic activity, restored ant-oxidant enzyme activity, decreased FBG level while calcium level and GLUT-4 expression was increased	[17]
Cardiac abnormalities in experimental atherosclerosis in rat	RF at 40 mg/kg together with CoRNS after hypolipidemic induction	SOD↑, CAT↑, GPx↑	CoRNS significant reduced lipid profile: LDL and cardiac enzymes (LDH, ALT, AST, ALP) with enhanced levels of HDL and antioxidants.	[18]
GTN-induced brain oxidative toxicity	RF at 100 mg/kg was treated before GTN-induced migraine	Lipid peroxidation↓, GSH↑, GPx↑	RF with selenium administration protected against GTN-induced brain oxidative toxicity by protecting brain MMCA activity, inhibiting free radicals and supporting the antioxidant redox system.	[19]
Migraine model	RF 100 mg/kg was treated before GTN-induced migraine	Lipid peroxidation↓, GSH↑	RF and vitamin E had a protective effect on the GTN-induced brain injury by inhibiting free radical production, regulation of calcium-dependent processes, and supporting the antioxidant redox system.	[20]

**Table 3:** Evidence of RF on the attenuation of reperfusion oxidative injury.

Model	RF Dose	Key findings	Conclusions	References
Stroke-induced brain damage (neuroprotection against excitotoxicity)	RF at 0.05–0.5 mM before glutamate or NMDA treatment	RF at the concentrations of 0.2, 0.3, and 0.4 mM were significantly neuroprotective against glutamate and NMDA.	RF ameliorate glutamate or NMDA-mediated excitotoxicity to CGCs	[21]
Cortical contusion injury (CCI)	RF treatment with 7.5 mg/kg, i.p; n = 7, 15 min after injury. A second dose was applied after 24h after injury.	Reducing brain edema formation, and inhibit GFAP+ expression, improve behavioral function.	Administration of RF following CCI of the frontal cortex improves recovery of function following injury	[22]
Cortical contusion injury (CCI).	RF was treated after CCI:a combination of 1 mmol/kg MgCl2 and 7.5 mg/kg RF	The combination of MgCl_2_ and RF improved the functional recovery while the half-dose combination did not.	RF and magnesium infusions improved functional recovery to a greater extent than either alone following a frontal cortical contusion injury in rats	[23]

RF can significantly protect against oxidant-mediated inflammatory injury in the lungs of Long-Evans rats caused by cobra venom factor or IgG immune complexes, or ischemia-perfusion [24]. RF has also been reported to have a protective role in focal ischemia with decreasing brain injury and oedema formation in rats [25]. RF also has cardio-protective effects in isolated rabbit cardiomyocytes, reducing elevated ferrylmyoglobulin induced by cardiac re-oxygenation damage. This effect is mediated by Flavin reductase [26].

### 2.3. Malaria Infection

Infections with malaria parasites may stimulate the immune system leading to ROS formation, which can attack the plasma membrane of the erythrocyte compromising its integrity [27]. RF reduced the level of methaemoglobin content, decreased food vacuole size and inhibited asexual parasite growth in erythrocytes infested with *P. falciparum* [28].

In addition, co-treatment of RF and Chloroquine tablets against malaria infection significantly increased the packed cell volume and haemoglobin (Hb) levels, but reduced lipid peroxidation, contributing to maintaining the redox integrity of cells, protecting them against ROS generated during the inflammatory response [29]. 

### 2.4. Immune System

RF activates phagocytic activity of neutrophils and macrophages, and stimulates the multiplication of neutrophils and monocytes [30]. It has also been shown that RF is important for the survival of macrophage RAW 264.7 cells. The reduction in RF concentration resulted in a decreased rate of cell proliferation [31]. A combined supplementation—consisting of RF, delta-tocotrienol and quercetin—improved the inhibition of serum tumor necrosis factor alpha (TNF-α) and nitric oxide (NO) levels in a chicken model [32]. 

However, RF administration affects neutrophil migration, inhibiting the infiltration and accumulation of activated granulocytes into peripheral sites, which may lead to a decreased inflammatory influx and, thereby, a decrease in inflammatory symptoms [33]. RF is a potential substance for use in virus inactivation, or as an adjuvant in chemo radiotherapy for cancer treatment because of its toxicological and photosensitizing attributes. RF suppressed T-cells infiltration and donor-reactive alloantibody formation during the early period after allotransplantation [34].

The pro-inflammatory transcription nuclear factor kappa B (NF-κB) is normally activated by degradation of inhibitory kappa B (IκB). When this occurs, NF-κB translocates to the nucleus and binds to specific promoter regions of genes encoding pro-inflammatory proteins. Proteasomes are key regulators of lipopolysaccharide (LPS)-stimulated inflammatory signalling pathways. RF, as proteasome inhibitor, possibly down-regulates the NF-κB activation initiated by ROS, which are the potent activators of a plethora of general pro-inflammatory cytokines such as interleukin-6 (IL-6), TNF-α, etc. Therefore, ultimately, as proteasome inhibitor RF suppresses the production of TNF-α and NO, and exerts anti-inflammatory effects by inhibiting NF-κB, activation. As was recently reported, RF may protect against multitude of age-associated diseases by inhibition levels of secretion of TNF-α, NO production, activation of NF-κB, and degradation [35].

In recent years, there has been much interest in the anti-nociceptive and anti-inflammatory effects of RF (Table 4). RF helps in reducing inflammatory nociceptive pain [36,37]. Several animal models have been used to study the possible role in anti-nociceptive and anti-inflammatory effects of RF. It has been indicated that RF could inhibit nociceptive responses induced by a number of inflammatory agents in a variety of structures. For example, RF inhibited the formalin-induced hind paw oedema [37]. Moreover, RF can improve the anti-nociceptive effect when combined with low-dose morphine in a formalin test model [38], as well as in a zymosan-induced peritonitis model [39]. The anti-inflammatory studies of RF on the zymosan-induced peritonitis model showed that RF effects were dependent on the time of administration and dose [40], as well as strain-specific differences in mice [38].

Another study showed that oral administration of RF reduced inflammation and nociception, which were induced by formalin via the activation of ATP-sensitive K^+^- channels or NO release [41]. 

Sepsis is attributed to a systemic inflammatory response to bacterial infection, leading to multiple system dysfunctions in the body. Many pro-inflammatory mediators play a role in immune reaction during septic shock with release of inflammation cytokines, e.g., TNF-α, IL-1, IL-6, chemokines (monocyte chemo attractant protein 1 (MCP-1), macrophage inflammatory protein (MIP-2)), and NO production, including high-mobility group protein B1 (HMGB1) [47]. The beneficial effects of RF are based on its anti-inflammatory capability. Several experimental animal models have shown that RF has a potent effect against mortality rates from LPS-induced septic shock, exotoxin and exotoxin-induced shock, Gram-positive and Gram-negative bacterial infections via reduction the elevated level of TNF-α, IL-1, IL-1β, IL-6, gamma interferon (IFN-γ), MCP-1, and 2 MIP-2 as well as NO levels [8,42,43]. 

In terms of the efficacy of treatment with RF in combination with antibiotics (ampicillin [48,49], azithromycin [50], Ciprofloxacin [49]) or amino acids [8] on septic arthritis, RF has been reported as causing an increase in the survival rate of mice [50].

Therefore, RF might represent a promising new therapeutic strategy for the treatment of sepsis and septic shock. However, to our knowledge, there are a limited number of studies that have examined the anti-inflammatory and anti-nociceptive effects of RF in humans. Most of the evidence that RF reduces inflammation and nociception responses comes from laboratory animal studies. It has been demonstrated that RF reduced the hepatocellular injury and hepatotoxicity induced by LPS through elevation of malondialdehyde (MDA) level and myeloperoxidase (MPO) activity, whereas a marked decrease in GSH content, GR and glutathione peroxidase (GPx) activity. Moreover, expression of inducible nitric oxide synthase (iNOS) and catalase (CAT) gene expression was improved via RF administration [45]. In addition, RF reduces mortality rates through the reduction of the expression and release of high-mobility group protein B1 (HMGB1); however, the effect of RF was time-dependent [46]. A high dose of RF was also indicated to decrease LPS-induced mortality by increasing the expression of heat shock protein 25 (HSP25) [44].

**Figure 4:**
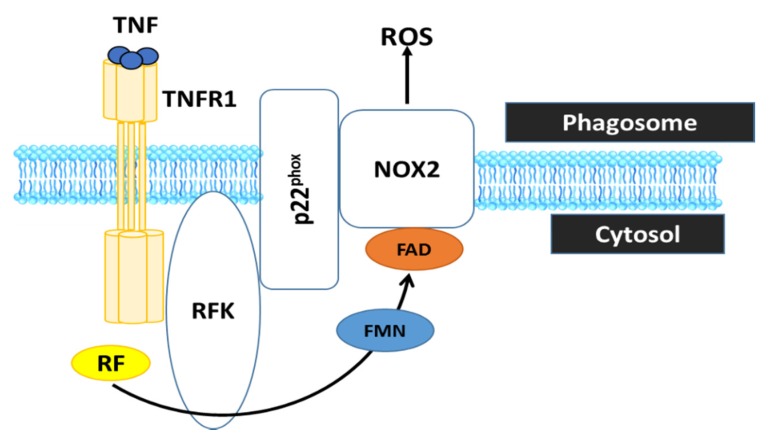
RF is converted by RFK into FMN and FAD, which is essential cofactor the phagocytic NADPH oxidase 2 (Nox2) to generate ROS. Therefore, RF deficiency is incapable of ROS production by the phagocyte Nox2, which is crucial to inactivate phagocytosed microbes and to regulate the inflammatory response in innate immune cells. TNF, tumor necrosis factor; TNFR1, tumor necrosis factor receptor 1.

RF, the precursor of FAD and FMN, is converted by riboflavin kinase (RFK) into FMN and FAD, which are essential cofactors of the phagocytic nicotinamide adenine dinucleotide phosphate (NADPH) oxidase 2 (Nox2). In particular, it has been shown that RF deficiency using conditional RFK knockout strains of mice impairs Nox2 priming. Such an effect may have implications for ROS production which impairs defence against *Listeria monocytogene* [51]. These data show that TNF priming of Nox2 represents a RF-dependent mechanism that is crucial for optimal ROS production in innate immune responses (Figure 4).

**Figure 5:**
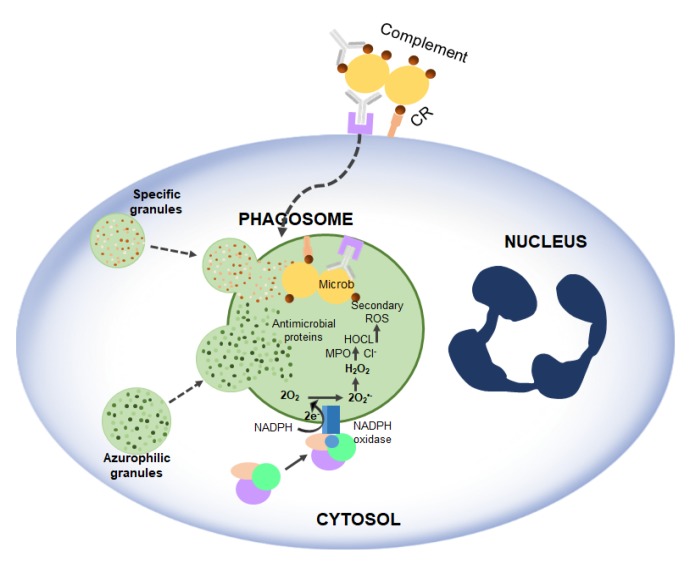
Activation of NADPH oxidase and microbicidal systems during phagocytosis. Complement and antibody receptors (CRs and FcRs) promote the uptake of micro-organisms by neutrophils, which, in turn, trigger the degranulation and production of ROS.

As mentioned above, RF plays a crucial role in Nox2. The experiment of Dey and Bishayi [49] showed that RF pre-treated macrophages lead to elevated levels of H_2_O_2_, O_2_^−^ as well as ROS and cause direct oxidative damage to many pathogens (Figure 5). RF along with antibiotics balances ROS and inflammatory cytokines and controls *S. aureus* infection by boosting murine macrophage function and regulates inflammation [49]. 

Wooley and Sebrell have shown that RF-deficient mice are more susceptible to fetal experimental pneumococcus infection than control groups [52].

The deleterious toxic effects of many toxic substances have been linked to an increased production of free radicals and/or ROS. Some reports have indicated that RF has a protective effect against mitochondrial toxicity and lipodystrophy when in combination with stavudine or both stavudine and lamivudine in animal models [53].

### 2.5. Photosensitizing Properties of RF

As a photosensitizer, RF induces oxidative damage to light-exposed tissue by the degradation of proteins, unsaturated lipids, folate, thiamine and other vitamins. This is due to the triplet-excitation state of RF resulting from light exposure, which can then be deactivated by phenolic and N-heterocyclic amino acids as well as their compounds. The deactivation of triplet-excited state RF by oxygen under aerobic conditions is faster than by anaerobic conditions through polyunsaturated lipids. Carotenoids do not appear to have deactivation properties for the triplet-excited state of RF, while vitamin E and plant polyphenols deactivate triplet-excited state RF and, therefore, protect proteins and lipids from degradation. On the other hand, polyphenols and carotenoids have protective effects on the degradation processes brought by triplet-excited state RF, however by different mechanisms [1]. Since RF acts as a photosensitizer it can be used with Long-wave-length ultraviolet irradiation in order to inactivate many pathogens like HIV, pseudorabies virus, West Nile virus, parvovirus, *E.coli*, and *Leishmanial protozoa* [54,55,56,57].

### 2.6. Cancer

RF deficiency has been implicated as a risk factor for cancer in general, although this has not been satisfactorily established and proved in humans [7]. There have been several articles reporting results of randomized controlled trials of RF on risk of cancer incidence. There has been an experimental finding that high folate (B vitamin) intake may reduce breast cancer risk in Chinese women [58]. The results of a study that has investigated the effect of RF on 786-O cells indicated that RF at the high dose can inhibit cell viability and has a significant reduction in the level of tumor necrosis factor receptor 1 (TNFR1) in 786-O cells [59]. In a study carried out by Machado et al. [60] showing a strong inhibitory effect of RF on melanoma metastasis formation in lung of animal model. In addition, RF is likely to decrease risk of colorectal cancer (CRC) among women. Methylenetetrahydrofolate reductase (MTHFR) is FAD-dependent and low intake of RF may disturb this enzyme activity and related with CRC [61]. Moreover, in post-menopausal women, intake of RF and vitamin B-6 related to a decreased CRC risk [62]. Few significant associations between the intake of RF and the risk of ovarian cancer were observed [63].

In female non-smokers, a higher intake of RF was correlated with a decrease the risk of lung cancer. The RF intake of 1.2 mg per day was associated with a lower risk of developing lung cancer compared to an intake of 0.52 mg per day [64]. There is a significant interest in RF in combination with thiamin, folate, and vitamin B12 from supplements and foods for a protective role in cervical carcinogenesis [65]. The importance of flavins in the folate metabolism as well as the combined protective effect of RF and folate is well known [66].

Further studies dealing with the RF doses are necessary to prove any efficacy in treatment such as cervical cancer or AIDS-related acidosis [3]. RF is associated with the inhibition in tumour growth in experimental animals and possibly in man; however, the regularity behind has not been discovered. The deficiency of RF increases the risk of cancer, while others propose an attenuating effect of some carcinogens [67]. A deficiency in RF can cause a disruption of the integrity of oesophageal epithelium and in some studies it is related to oesophageal cancer [68], while others relate to an increased susceptibility to cancer [69]. 

**Figure 6:**
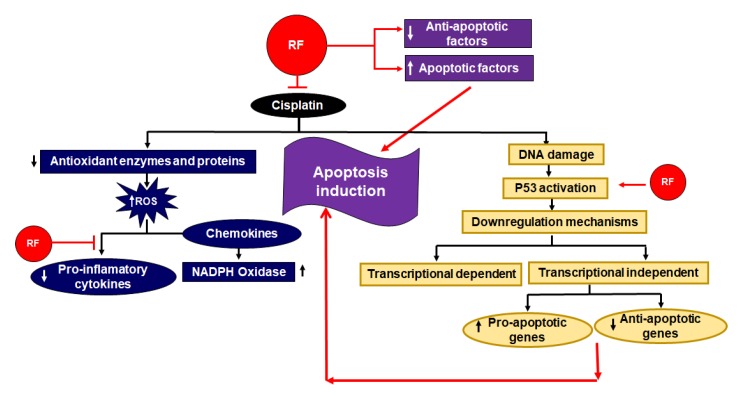
Role of RF as an adjuvant in cisplatin based chemo radiotherapy [75]. RF stimulates apoptotic factors and downregulates anti-apoptotic factors. Additionally, it activates p53, which also results in an amplification of apoptosis. The inhibitory effects of RF in respect to the deteriorate effect of cisplatin are also shown. On the one hand, it inhibits the downregulation of antioxidant enzymes and proteins; on the other, RF downregulates pro-inflammatory cytokines.

A high dose of RF intake showed a reverse effect of hepato-carcinogens in rodents [70]. One study proposed that a moderate amount of RF can initiate the extrinsic pathway of apoptosis. Higher levels of RF can trigger further cell death mechanisms like the intrinsic pathway by the action of down regulation of anti-apoptotic factors and by the upregulation of apoptotic factors (Figure 6) [71]. 

The overall outcomes in the available studies are optimistic but not yet convincing. There are further studies to be made to obtain more knowledge about the role and mechanism of action of vitamins and other micronutrients in terms of prevention and treatment of cancer. On the other hand, RF is considered to have an enormous capability to be used in improving the chemotherapeutic potential of anticancer drugs [72].

In addition, RF laurate has protective and therapeutic properties against radiotherapy-induced toxicity on human embryonic lung fibroblasts (Helf cells) [73].

The oxidative damage of cells from chemotherapeutic drugs can be ameliorated by RF administration. RF can improve the chemotherapeutic potential of major anticancer drugs like carbon tetrachloride (CCL_4_) via decreasing the hepatic oxidative stress and the release of pro-inflammatory cytokine TNF-α from leukocytes in CCl_4_-induced hepatic injury [74].

### 2.7. Migraine

Currently it is not clear how RF contributes to migraine prevention. However, the reduction in oxygen metabolism due to a mitochondrial dysfunction may play a role in migraine prevention since an increase in RF concentration might enhance the brain mitochondrial functions [76]. Studies have indicated a positive influence of RF in migraine prophylaxis, as RF is considered to play a potential role due to its attributes in the sense of safety, toleration and as an economically reasonable substance [77]. A clinical study reported that RF is an effective and low-cost prophylactic treatment in children and adolescents suffering from migraine [78]. In a study of migraine sufferers, RF combined with feverfew and magnesium showed a significant reduction in number of migraine attacks, migraine days and migraine index in the 3-month trial. When treatment response was compared, no significant differences were seen between the groups [79]. It has also been demonstrated that RF supplemented with magnesium and Q10 ameliorates migraine symptoms [80].

### 2.8. Cataract

Cataract formation is a result of protein aggregation which causes the lens to become cloudy. RF intake from food and supplements was associated with decreased risk of nuclear lens opacities [81]. Cataract formation in the general public seemed not to be associated with RF deficiency while in the elderly it might be increased due to a RF deficiency [82]. High dose of RF, 400 mg/d, appears to have a preventive effect or some beneficial effects on the development of age-related cataracts [3]. RF concentration influences the GSH concentration in the lens, while GSH protects the lens against oxidative damage and cataract development. In total, 80% of the cataract patients showed a shortage of RF [83]. 

### 2.9. Premenstrual Syndrome (PMS)

Premenstrual syndrome (PMS) is a condition that refers to a complex of physical and psychological symptoms which occur during the luteal phase of the menstrual cycle and disappear when menstruation starts. An increased intake of RF from food sources was associated with a decrease in the risk of PMS. The intake of 2.52 mg RF per day was observed to lead to a 35% lower risk of developing PMS compared to the intake of 1.38 mg per day [84].

### 2.10. Bone

Osteoporosis is a systemic skeletal disease. One of the most important factors to influence risk of fracture is vitamin intake. RF had an additive effect on ascorbate and β-glycerophosphate-induced osteoblast differentiation of MC3T3-E1 including intensifying G0/G arrest in pre-osteoblast cells [85]. 

### 2.11. Neuropathy

Indications have been made for RF being important in the early postnatal development of the brain [86]. The reduction in RF uptake and decrease in RF transporter protein (RFVT2) expression due to mutations in SLC52A2 gen has been illustrated. Patients with these mutations have shown significant and sustained clinical and biochemical improvements after high-dose oral RF therapy [87]. Moreover, RF co-treatment with selenium or vitamin E can have a protective effect on brain and microsomal membrane Ca^2+^-ATPase (MMCA) and oxidative damage caused by glyceryl trinitrate (GTN)-induced headaches in rat models. RF and selenium (SE) can decrease lipid peroxidation (LP) levels and increase glutathione and reduced glutathione levels in microsomal brains. The co-administration of RF and vitamin E induced a decrease in calcium and a decrease in brain and microsomal lipid peroxidation levels [19,20]. The brain and microsomal lipid peroxidation levels (LP) have been found to be higher in the GTN-induced rats with migraine headaches compared to healthy rats [19]; whereas SE orally pre-treated rats with or without RF were protected against GTN-induced brain oxidative toxicity, by inhibiting free radicals and MMCA activity and supporting the antioxidant effect of RF [19]. It has been demonstrated that RF plays a protective role in a time- and dose-dependent manner against excitotoxicity of cerebellar granule neurons induced by glutamate/NMDA in C57/B strain mice [21]. It has been shown by authors [22] that RF may have therapeutic potential for the treatment of traumatic brain injury. RF administration after frontal cortical contusion injury (CCI) in rats improved behavioural outcomes, reduced the number of glial fibrillary acidic protein (GFAP^+^) astrocytes, reduced brain oedema formation and reduced lesion size [22]. In addition, it is also suggested to combine RF and MgCl_2_ infusions, to be more effective in improving functional recovery after bilateral and unilateral cortical CCI in rats [23].

### 2.12. Anemia

RF contributes to blood cells formation as it plays a role in erythropoiesis, improves iron absorption and helps in the mobilization of ferritin from tissues [88]. The concentration of hemoglobin was able to be increased by RF supplementation. In an animal model, RF was also shown to enhance iron absorption [89], while RF deficiency increases the rate of gastrointestinal loss of iron and decreases the mobilization of iron from its stores [90].

A positive relation between RF intake and anemia in women, especially those below 50 years, was observed. Furthermore, a significant association between RF and iron intake in correspondence to the risk of anemia was detected. There have been cross-sectional studies to illustrate the link between RF intake and anemia, while prospective population studies are limited [91]. 

### 2.13. Diabetes Mellitus

Oxidative Stress is one of the major factors in the pathogenesis of type-2-diabetes mellitus (T2DM). The dietary intake of RF might lead to a reduction in diabetic complications because of the reduction in inflammatory processes triggered by oxidative stress and ROS formation. RF is indicated to have a positive effect on blood sugar. It plays a role in the absorption of sugar from the intestine and reduces hyperglycemia. It enhances the state of hyperglycaemias by increasing glucose uptake in skeletal muscles and white adipose tissue, as well as significantly ameliorating oxidative stress, tissue damage and cellular DNA damage in T2DM in mice. The ability of antioxidants to inhibit injury has raised the possibility of new therapeutic treatment for diabetic heart diseases. RF has shown promising beneficial effects on heart failure in type I diabetic cardiomyopathy in rats [16,17]. 

### 2.14. Cardiac Abnormalities

RF as a single compound has shown promising results for protective actions in terms of cardiac abnormalities. Cardiac abnormalities could be avoided or reduced by using RF. RF may be combined with other compounds to have additive beneficial effects. RF combination with coenzyme Q10, niacin, selenium as CoRNS and Emblica officinalis has a protective effect on cardiac abnormalities in experimental atherosclerosis [18]. RF also reduces subsequent acute rejection and early graft oxidant stress significantly after allotransplantation and coronary allograft vasculopathy as results of cardiac ischemia-reperfusion. Pre-treatment with RF in a murine heart transplantation model can protect tissues from oxidative damage by decreasing myocardial lipid peroxidation, leukocyte infiltration, cytokine production, and cardiac allograft vasculopathy [34].

### 2.15. Hypertension

Hypertension is considered to be the leading risk factor for mortality worldwide [92]. It is estimated to be responsible for 8 million premature deaths per year [93]. A study has proposed an association of RF intake and blood pressure modulation. In animal studies, RF as supplementation did not provoke the side effects, but did provoke a significant systolic blood pressure reduction which was not age dependent between young and adult rats [94].

## 3. Side Effects of Lack or Excess of RF 

RF deficiency has been comprehensively reviewed elsewhere [7]. RF is required in many oxidation-reduction reactions and, therefore, RF deficiency may affect many systems. RF is considered to be one of the most common vitamins with deficits in people of developing countries, particularly the ones with rice as their staple food. In those countries, important RF sources like milk and meat are not sufficiently consumed [95]. However, ariboflavinosis is not a common deficiency in most societies. RF deficiency is only measurable by quantifying the vitamin concentration in body fluids like blood plasma, serum, etc. [96] (Table 1). 

**Figure 7:**
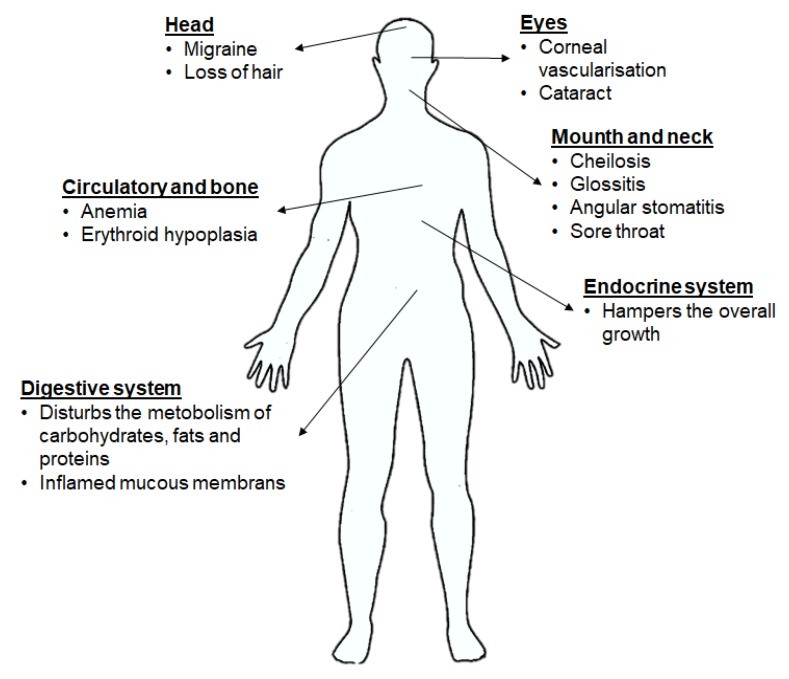
Implications of RF deficiency on health [75].

Ariboflavinosis in humans causes various symptoms like sore throat, hyperaemia, oedema of the oral and mucous membranes, cheilitis and glossitis, hair loss, inflammation of skin, cataract development, migraine and a decrease in Hb. RF deficiency has shown an influence on the iron absorption, metabolism of tryptophan, mitochondrial function, brain and the metabolism of other vitamins. The Food and Nutrition Board 1998 published that a balanced diet meets the recommended intake of 1.4 mg RF per day for an adult man. A study observed that approximately 60% of elderly people were at risk for RF deficiency [97]. The shortage of RF could also be a consequence of the use of some drugs, alcohol consumption, increased need of RF due to physiological conditions like pregnancy or breastfeeding or childhood etc. (Figure 7) [7,75,88].

RF deficiency rarely occurs alone and is usually part of generalized vitamin B deficiency because of a poor diet or malabsorption. Oral supplementation of RF alone is poorly absorbed, with only 15% of bioavailability. RF can be destroyed by UV light exposure; UV therapy in infants with hyperbilirubinemia could cause RF deficiency [3]. 

## 4. Mechanism of Antioxidant Protection

**Figure 8:**
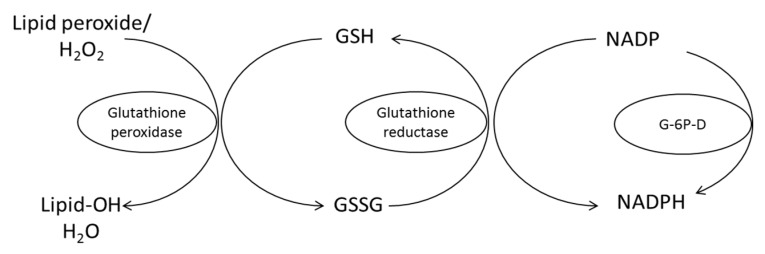
Conversion oxidized glutathione (GSSG) to the reduced form (GSH) by glutathione reductase requires RF in the FAD co-enzyme form for its activity. G-6P-D, glucose-6-phosphate dehydrogenase [98].

RF has an antioxidant function that can destroy ROS [10]. The powerful antioxidant activity is derived from its role as precursor to FMN and FAD as important coenzymes required by a number of enzymes involved in oxidative metabolism, particularly glutathione oxidase. On the other hand, GR—which is a free radical scavenger—also needs FAD as a cofactor. The protective role of RF against various diseases has been described previously [98]. Several studies showed that RF is effective to reduce ROS in various diseases including anti-aging and it also helps to ameliorate the toxic effect of drugs in various treatments. Many studies investigated the antioxidant effect of some vitamins such as vitamin E, vitamin C, carotenoids and RF. RF effective mechanism has not been completely investigated. It acts as a coenzyme for redox enzymes in FAD and FMN forms and can have a potential effect on oxidative stress reduction as an antioxidant by prevention of lipid peroxidation and by attenuation of reperfusion oxidative injury. GR requires RF for its activity to convert GSSG to the GSH. FAD transports hydrogen from NADPH to GSSG to convert it into the GSH form (Figure 8). GSH acts intracellular as an endogenous antioxidant and deactivates ROS. Glutathione deactivates peroxides such as hydroperoxides by the action of GPx from GSH to lipid peroxide and produce GSSG and alcohol. Therefore, RF deficiency leads to an increase in lipid peroxidation.

## 5. Perspective Use of RF in Complementary Medicine: Administration via Functional Food and Nanocapsules

### 5.1. RF in Food

**Figure 9:**
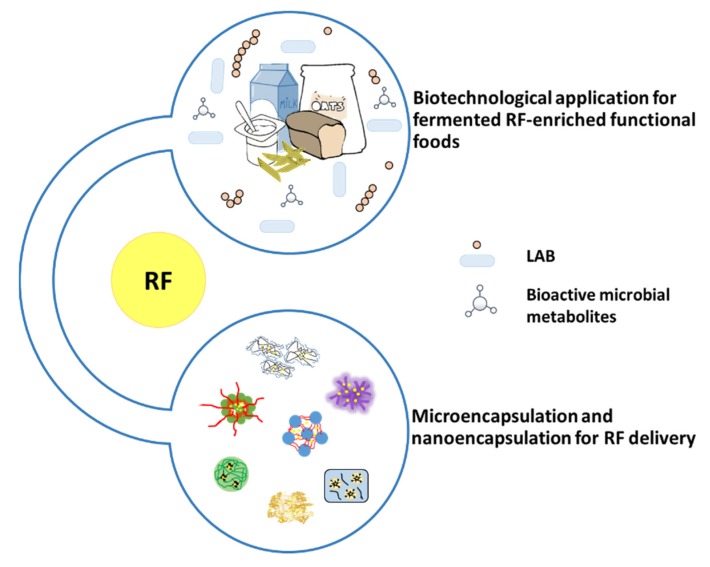
RF in functional food and encapsulation products.

Biotechnological application is used for fermented RF-enriched functional foods. Lactic acid bacteria, can synthesize or utilize during food fermentation, are considered for the production of vitamin-enriched food (Figure 9). Using *L. lactis* to produce fermented milk overcomes ariboflavinosis in a RF-deficiency rat model [99]. *L. plantarum* was used to produce fermented RF-enriched sourdough bread and pasta [100], soymilk [101], oat-based food [102]. *L. fermentun* was found to produce RF to fortify bread [103]. Furthermore, *P. freudenreichii* lead to an increase in RF content in yoghurt with *P. freudenreichii* [104] or fermented milk with this *Propionibacterium* species reverted ariboflavinosis in mouse [105].

### 5.2. RF Encapsulation

RF is sensitive to light, especially at high temperatures and in alkaline conditions [106]. In food process modifications, fortification or encapsulation has the potential to alter the stability of RF in food (Figure 9). Therefore, to maintain the level of this vitamin, these factors need a consideration for its stability. Encapsulation has been proposed as a solution for its chemical instability [107,108,109]. 

Micro/nano-encapsulation was used to solve RF chemical instability. Numerous encapsulating techniques are currently implemented and selected according to the physical and chemical properties of core materials and encapsulating agents. Some example methods used for RF micro/nano-encapsulation are shown in Table 5.

Blank whey microbeads manufactured using a cold-set gelation process, were placed in solutions of RF [109]. As the volume of microbeads added to the solution was increased, the uptake of the compounds increased, to a maximum of 57%. The release kinetics of RF can be fitted by a pseudo first-order kinetic model very well. In addition, in vitro and in vivo release of RF from microbeads was evaluated [110]. In the first hour, the release of RF from dried microbeads was decreased in comparison to wet microbeads in gastric and intestinal settings, with 58% and 34%, respectively. The in vivo release of RF from dried microbeads—microbeads fed to piglets were shown to be only partially degraded—demonstrating good resistance to gastrointestinal degradation after ingestion. These aspects led research interests towards dried whey microbeads able to effectively deliver these substances for oral delivery to the intestine.

Hydrogels of soy protein isolate (SPI) were prepared via high intensity ultrasound (HIU) and were cross-linked by transglutaminase (TGase) [111]. Ultrasound treatment resulted in decreasing the RF release from TSGRs in simulated intestinal fluid (SIF) and simulated gastric fluid (SGF) with or without digestive enzymes. In addition, both encapsulation efficiency and gelation yield increased after ultrasound treatment. 

Azevedo et al. loaded RF in alginated-chitosan NPs prepared by ionic gelation [112]. The average size of nanoparticles with RF was 104 ± 67 nm (PDI 0.32 ± 0.07) with a Zeta-potential of −29.6 ± 0.1 mV. The nanoparticles showed entrapment efficiency (EE%) and loading capacity (LC) values of 56 ± 6% and 2.2 ± 0.6%, respectively. The release profiles were affected by polymeric relaxation. In addition, the stability of alginate–chitosan was measured over a five-month period at 4 °C in solution.

**Table 5:** Techniques and characteristic of micro/nanoencapsulation of encapsulated RF.

Encapsulation Techniques	Wall Material	Illustration of Characteristics	Purpose	Size	References
Cold-set gelation	Whey protein isolated	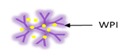	Proofing suitability of encapsulation system for intestinal delivery using in vitro and in vivo models	1.8 mm	[11]
Cross-linking of HIU-treated SPI with TGase	Soy protein isolated (SPI)	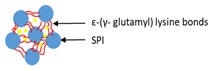	Demonstrating of HIU-treated SPI–TGase cold gel for longer retention in the gastrointestinal system	3 mm	[111]
Ionotropic gelation	Alginate/chitosan nanoparticles	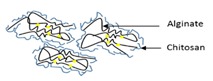	Establishing of alginate/chitosan nanoparticle for controlled release in different temperature and pH conditions	119.5 ± 49.9 nm	[112]
Ultrasonication	Soy protein/dextran nanogel	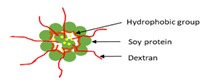	Providing basic design of soy protein/dextran nanogel for effective and suitable carriers for bioactive compounds	143.3 nm	[113]
Bioconjugation	Phenylalanine ethyl ester–alginate conjugated (PEA)	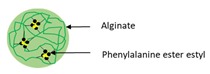	Illustrating a sonication method of self-assembled nanoparticles formed by PEA conjugate without cytotoxicity against cell lines	200 nm	[114]
Supercritical fluid technology	Fully hydrogenated canola oil	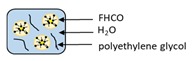	Evaluating surfactant and molecular weight of stabilizer from supercritical fluid technology for development of solid lipid nanoparticles	104 ± 5.7 nm	[115]
Coprecipitation-Crosslinking-Dissolution technique (CCD-technique)	Human serum albumin	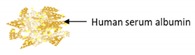	Demonstrating a simple coprecipitation method of albumin submicron particles with good biocompatibility	900 ± 1000 nm	[116]

Jin et al. [113] encapsulated RF using modified soy protein/dextran with the range of 143.3 nm size. The EE% and LC values of nanogels were up to 65.9% and up to 12%, respectively. The release rate of RF was examined in SIF and SGF. Nanogels have a better sustained release in SGF than in SIF.

Recently, Zhang et al. [114] produced phenylalanine ethyl ester–alginate conjugate (PEA) using EDC–NHS coupling reaction. Nano-encapsulated RF in PEA was synthesized via sonication. It was observed that the size of produced nanoparticles was 200 nm, meanwhile the LC and EE% were 3.53% and 91.48%, respectively. RF-loaded PEA-nanoparticles displayed pH-dependant release trend. By declining pH, the release rate of RF decreased. Moreover, the cytotoxicity of these particles to Caco-2 cells was used to investigate. There is no significant cytotoxicity against cell line in a wide range of concentration.

Solid lipid-based nanoparticles loaded with RF were prepared using fully hydrogenated canola oil-based lipids, sodium lauryl sulfate and polyethylene glycol as surfactant and stabilizer, respectively [115]. An LC of 12% to 48% RF was reported while bioactive loads varied from 0.09 to 0.73 mg/g, with particle sizes in the range of 105 nm.

The Co-precipitation Crosslinking Dissolution technique (CCD-technique) was employed in the presence of human serum albumin (HSA) and RF to produce albumin submicron particles. The uniform peanut-like particles showed a narrow size distribution in the range of 0.9 to 1 µm and a negative zeta-potential. The RF-albumin submicron particles revealed a good hemocompatibility [116].

## 6. Conclusions

RF is an important vitamin in the protection and treatment of various medical conditions. However, due to the few available studies on humans it is essential to have more data in terms of human clinical trials to conclude precise evidence and dosage information as recommendations for the treatment or prophylaxis of many diseases and conditions. The use of RF in fighting pathogens using UV-Light is a motivating approach, since it does not go along with adverse side effects. Through the consumption of functional RF-enriched foods, many diseases could possibly be prevented according to the currently available scientific evidence.

## Figures and Tables

**Table 1:** Flavin content of selected human body fluids (mol L^−1^) and organs (mol per kg of dry matter) [1].

	Riboflavin	FAD	FMN
Skin	7.6 × 10^−6^	—	—
Cerebral cortex	7.2 × 10^−6^	—	—
Myocardium	3.2 × 10^−5^	—	—
Pectoral muscle	7.2 × 10^−6^	—	—
Aortic tissue	4.8 × 10^−7^	9.7 × 10^−7^	2.2 × 10^−7^
Erythrocyte	1.4 × 10^−7^	4.3 × 10^−7^	2.8 × 10^−8^
Plasma	1.0 × 10^−8^	6.3 × 10^−8^	7.5 × 10^−9^
Eye-fluid	4.5 × 10^−6^	—	—

**Table 4:** Antinociception and anti-inflammation effects of RF in animal model.

Animal Model	RF Doses/Models	Major Outcome	References
**Inflammation-Related Pain**			
Acetic acid-induced abdominal constructions, formaldehyde-induced nociceptive response and hot-plate models in mice	RF at 3–100 mg/kg i.p. injection 1 h before acetic acid-induced model, RF at 6 or12 mg/kg i.p. injection 1 h before formaldehyde-induced nociceptive response, and RF at 50 mg/kg i.p. injection 1 h before formaldehyde-induced hindpaw edema	A dose-dependent RF inhibited the nociceptive response produced by acetic acid. Pre-treatment RF remarkably reduced the acute nociceptive response induced by formaldehyde in the second phase, but not in the hot-plate model. RF moderately inhibited formaldehyde-induced hindpaw edema.	[37]
Formalin-induced and carrageenan-induced paw edema, and spinal nerve ligation models in rat	RF at 1–50 mg/kg oral administration 30 min before formalin test and 6.25–150 mg/kg immediately after carrageenan injection	Second phase treatment with RF produced a significant dose-dependent inhibition in flinching behavior produced by formalin and RF at 25 mg/kg dose had peak antinociceptive effect in formalin-induced model. RF reduced hyperalgesic effect, highest effect at 75 mg/kg dose. In addition, a dose- and time-dependent RF treatment reduced by carrageenan-induced edema, but not tactile allodynia in the spinal nerve ligation models. Moreover, antinociceptive effect of RF can be reversed by glibenclamide and NG-L-nitro-aeginie methyl ester.	[41]
Formalin-induced nociceptive response, carrageenan-induced paw edema, LPS-induced febrile response, and cotton pellet-induced formation of fibrovascular tissue models in rat	RF at 25, 50, 100 mg/kg i.p. injection 30 min before formalin-induced nociceptive response, carrageenan-induced paw edema, RF at 50 or 100 mg/kg immediately or 2 hr after LPS-induced the febrile response, and RF at 50 or 100 mg/kg i.p. 7 days after s.c. implantation of a cotton pellet-induced fibrovascular tissue	RF inhibited the nociceptive response in the mouse formalin test, markedly in second phase. RF was dose-dependently reduced the mechanical allodynia and the paw edema induced by carrageenan and inhibited the fever induced by LPS. Moreover, the formation of fibrovascular tissue induced by s.c. implant of a cotton pellet was inhibited. Therefore, RF prevents prolonged inflammatory response.	[36]
Zymosan-induced peritonitis in Swiss mice	RF at 20, 50, 100 mg/kg i.p. injection 30 min before zymosan administration; RF at 50 mg/kg in combination with 5 mg/kg morphine	RF at 50 and 100 mg/kg induced antinociceptive-related body writhes and RF at 100 mg/kg dose suppressed intraperitoneal PMN influx. On the other hand, RF co-injected with morphine at low dose had antinociceptive effect and also reduced levels of proinflammatory cytikines such as TNF-α, IL-12p07, and IFN-γ according to RF dose and the time of injection.	[39]
**Anti-Inflammatory Effect**			
Toxin-induced shock (LPS-induced shock and *S. aureus* enterotoxin B (SEB)-induced shock) and bacterial infection in mice	RF at 2.5, 5, 10, and 20 mg/kg bolus injection 6 h after LPS injection or SEB–D-galactosamine injection. RF at 2.5, 5, 10, 20 mg/kg 1 day before *E. coli* inoculation or 1 and 2 days after *S. aureus* inoculation.	RF decreased the mortality of endotoxin- and exotoxin-induced shock, gram-negative and gram-positive bacterial infection including long-term treatment. In addition, RF reduced levels of plasma inflammatory cytokines, including TNF-, IL-1β, IL-6, IFN-γ, MCP-1, MIP-2, and NO level. Moreover, co-administration RF with APC ameliorated survival rate of toxin-induced shock.	[42]
LPS-induced shock model and bacterial infection model in mice	RF at 2.5, 5, 10, 20, 40, and 80 mg/kg/6h i.v. infusion after 6 h LPS injection. RF at 80 mg/kg/6 h after 1 h *E.coli* infection or RF at 20, 40, 80 mg/kg/6 h after 1 h *S.aureus* infection.	RF protected mice against the mortality in both toxin shock and infection models, but RF reduced only the level of IL-6 and NO in plasma. In addition, RF decreased the elevation of TNF-α, IL-1β, MPC-1, IL-6, and NO level in plasma.	[8]
LPS-induced shock model in mice	RF at 2.5 or 10 mg/kg for 6 h continuous i.v bolus administration with or without aminolevane^®^ or single dose injection with or without amino acids or valine after 6 h LPS injection.	RF at 10 mg/kg administered continuously for 6 h reduced morbidities on LPS- induced shock model, and was better with aminolevane^®^ combination treatment. RF treatment in combination with tryptophan, isoleucine, proline, threonine, alanine or valine had improved the survival rate, but only valine was significantly effective. Moreover, RF reduced IL-6, lactic acid level, but increased glucose level.	[9]
Endotoxin-induced shock in mice	RF at 20 mg/kg i.v. administered after 6 h LPS injection	RF decreased the number of IL-6 and MIP-2 and NO levels in plasma. RF also reduced IL-6 and MIP-2 levels in lung, but inhibited only MIP-2 level in liver. However, RF reduced IL-6 mRNA expression in lung, but MIP-2 mRNA expression was inhibited in liver and kidney. Additionally, iNO expression was inhibited by RF.	[43]
Olive oil-triggered paw swelling and collagen-induced arthritis models in mice	RF at 20 mg/kg i.p. administration before oil injection or after collagen-induced arthritis	RF inhibited the paw swelling induced by olive oil, affecting a reduction in neutrophil-dependent reaction. However, RF could not inhibit delayed type hypersensitivity reactivity and collagen II arthritis.	[33]
LPS-induced shock model in mice	RF at 1 and 10 mg/kg i.p. injection at 2 and 0 h before LPS administration	RF significantly suppressed the LPS-induced lethality in mice septic shock model and RF have protective effect through up-regulated the expression of HSP25 in the lung and heart.	[44]
Zymosan-induced peritonitis in different C57BL/6J, BALB/c and CBA mice strains	RF at 50 mg/kg i.p. co-injection with zymosan (40 mg/kg)	RF co-treatment with zymosan reduced pain symptoms. Anti-inflammatory effects of RF are strain-specific manner. Particularly, peritoneal leukocytes (PTL) accumulation was inhibited in BALB/c and CBA strains, but was prolonged in C57BL/6J strain. The expression of iNOS was delayed (C57BL/6J) or inhibited (BALB/c and CBA) in PTL lysates as well as the prolonged (C57BL/6) or inhibited (BALB/c) intraperitoneal accumulation of MMP-9.	[38]
Zymosan-induced peritonitis in Swiss mice	RF at 0, 20, 50, or 100 mg/kg by co-injection, pre-injection or post-injection in zymosan-induced peritonitis	RF itself induced nociceptive-related body writhes, but effectively reduces zymosan-induced writhing response on influence of pre-injection or post- injection. RF also reduced the evaluation number of PLTs, mainly PMN and an increase in inflammation-related cytokines and MMP-9 with dose- and administration time-dependent effect.	[40]
LPS-induced acute lungs injury in rat	RF at 30 mg/kg, p.o. for 7 days before LPS intranasally (i.n.)	RF reduced interstitial edema, hemorrhage, infiltration of inflammatory PMNs, and destruction of lung parenchyma as well as decreased the iNOS level, but enhanced GSH, GR, GRx, and CAT expression.	[45]
Zymosan-induced inflammation in mice and in vitro macrophages	RF at 50 mg/kg i.p. injection 30 min either before zymosan, together with zymosan, or 2, 4, 6 h after i.p. zymosan injection.	RF causes both the inhibition of expression and release of HMGB1 in time-dependent manner.	[46]

## Abbreviations

RF	Riboflavin
FMN	Flavin mononucleotide
FAD	Flavin adenine dinucleotide
SOD	Superoxide dismutase
ROS	Reactive oxygen species
GR	Glutathione reductase
GPx	Glutathione peroxidase
GSSG	Oxidized glutathione
GSH	Reduced glutathione
OGD	Oxygen glucose deprivation
LDH	Lactate dehydrogenase
Hb	Haemoglobin
TNF-α	Tumor necrosis factor alpha
NO	Nitric oxide
NF-κB	Nuclear factor factor kappa B
IκB	Inhibitory kappa B
LPS	Lipopolysaccharide
IL-6	Interleukin-6
MCP-1	Monocyte chemo attractant protein 1
MIP-2	Macrophage inflammatory protein ()),
HMGB1	High-mobility group protein B1
MDA	Malondialdehyde level
MPO	Myeloperoxidase
CAT	Catalase
HSP25	Heat shock protein 25
RFK	Riboflavin Kinase
NADPH	Nicotinamide adenine dinucleotide phosphate
Nox2	NADPH oxidase 2
TNFR1	necrosis factor receptor 1
CRC	Colorectal cancer
MTHFR	Methylenetetrahydrofolate reductase
CCL_4_	Carbon tetrachloride
PMS	Premenstrual syndrome
RFVT2	RF transporter protein 2
MMCA	Microsomal membrane Ca2+-ATPase
GTN	Glyceryl trinitrate
SE	Selenium
CCI	Cortical contusion injury linear dichroism
GFAP+	Glial fibrillary acidic protein
T2DM	Type-2-diabetes mellitus
PTL	Peritoneal leukocytes
SPI	Soy protein isolate
HIU	High intensity ultrasound
TGase	Transglutaminase
SIF	Simulated intestinal fluid
SGF	Simulated gastric fluid
EE%	Entrapment efficiency
LC	Loading capacity
PEA	Phenylalanine ethyl ester–alginate conjugate
CCD-technique	Coprecipitation-Crosslinking-Dissolution technique
